# Investigating the cultural and contextual determinants of antimicrobial stewardship programmes across low-, middle- and high-income countries—A qualitative study

**DOI:** 10.1371/journal.pone.0209847

**Published:** 2019-01-16

**Authors:** Esmita Charani, Ingrid Smith, Brita Skodvin, Anne Perozziello, Jean-Christophe Lucet, François-Xavier Lescure, Gabriel Birgand, Armel Poda, Raheelah Ahmad, Sanjeev Singh, Alison Helen Holmes

**Affiliations:** 1 NIHR Health Protection Research Unit in Healthcare Associated Infections and Antimicrobial Resistance, Imperial College London, London, United Kingdom; 2 Department of Essential medicines and Health Products, World health Organization, Geneva, Switzerland; 3 Norwegian advisory unit for Antibiotic use in Hospitals, Haukeland University Hospital, Bergen, Norway; 4 Assistance Publique-Hôpitaux de Paris (AP-HP), Bichat-Claude Bernard Hospital, Infection Control Unit, Paris, France; 5 IAME, UMR 1137, INSERM, Université Paris Diderot, Sorbonne Paris Cité, Paris, France; 6 School of Medicine, University Hospital Souro Sanou, University of Bobo Dioulasso, Bobo Dioulasso, Burkina Faso; 7 Department of Medicine, Amrita Institute of Medical Sciences, Amrita University, Kerala, India; University of Georgia, UNITED STATES

## Abstract

**Background:**

Most of the evidence on antimicrobial stewardship programmes (ASP) to help sustain the effectiveness of antimicrobials is generated in high income countries. We report a study investigating implementation of ASP in secondary care across low-, middle- and high-income countries. The objective of this study was to map the key contextual, including cultural, drivers of the development and implementation of ASP across different resource settings.

**Materials and methods:**

Healthcare professionals responsible for implementing ASP in hospitals in England, France, Norway, India, and Burkina Faso were invited to participate in face-to face interviews. Field notes from observations, documentary evidence, and interview transcripts were analysed using grounded theory approach. The key emerging categories were analysed iteratively using constant comparison, initial coding, going back the field for further data collection, and focused coding. Theoretical sampling was applied until the categories were saturated. Cross-validation and triangulation of the findings were achieved through the multiple data sources.

**Results:**

54 participants from 24 hospitals (England 9 participants/4 hospitals; Norway 13 participants/4 hospitals; France 9 participants/7 hospitals; India 13 participants/ 7 hospitals; Burkina Faso 8 participants/2 hospitals) were interviewed. Across Norway, France and England there was consistency in ASP structures. In India and Burkina Faso there were country level heterogeneity in ASP. State support for ASP was perceived as essential in countries where it is lacking (India, Burkina Faso), and where it was present, it was perceived as a barrier (England, France). Professional boundaries are one of the key cultural determinants dictating involvement in initiatives with doctors recognised as leaders in ASP. Nurse and pharmacist involvement was limited to England. The surgical specialty was identified as most difficult to engage with in each country. Despite challenges, one hospital in India provided the best example of interdisciplinary ASP, championed through organisational leadership.

**Conclusions:**

ASP initiatives in this study were restricted by professional boundaries and hierarchies, with lack of engagement with the wider healthcare workforce. There needs to be promotion of interdisciplinary team work including pharmacists and nurses, depending on the available healthcare workforce in different countries, in ASP. The surgical pathway remains a hard to reach, but critical target for ASP globally. There is a need to develop contextually driven ASP targeting the surgical pathway in different resource settings.

## Background

Research in tackling antimicrobial resistance (AMR) and implementing antimicrobial stewardship is necessary from different cultures, economies and healthcare organisations. There are increasing calls for identifying global solutions to the threat of AMR [1,2]. It is only when we know what the challenges are across the spectrum of healthcare globally that we can start finding contextually fit solutions. It is often argued that quality in healthcare should be universal, and it should be accountable to the same standards worldwide. Likewise, for antimicrobial stewardship programmes (ASP), a shared agreement of its central principles is necessary for it to be implementable across different countries and economies[3]. The first step to doing this is to understand what the contextual and cultural determinants are in different countries and how they can be addressed. Decision-making in healthcare is complex and contextually driven with multiple actors and actions[4]. This complexity is evident in antibiotic decision-making where different priorities and contextual factors influence behaviours[5–9]. The influence of hierarchies and peer influence on antibiotic prescribing in hospitals[5,10], with the need for tailoring interventions to the context in which they are to be implemented and ensure local ownership[11]. Countries however are at different stages of implementing ASP[12–14], yet, most research and evidence for ASP continues to be from high income countries[15–17]. The latest Cochrane systematic review of interventions to improve antimicrobial prescribing, included 221 studies[17] of which, 183 (83%) were from Europe and North America. One of the findings of the Cochrane review was that few interventions include behavior change or behavior theory, and globally reported variation in antibiotic prescribing cannot be resolved without an appreciation and understanding of the social influences in antibiotic decision-making[18]. There remains a gap in the evidence base on the effective means of 1) influencing antibiotic prescribing behaviours and; 2) effective and sustainable assimilation of stewardship interventions into existing healthcare frameworks. There is now a growing interest in low- and middle-income countries (LMIC) to implement programmes to control and optimise antimicrobial use[13,14,19,20]. These efforts are also supported by international reports highlighting the urgent need to tackle AMR on a global scale[21,22].

ASP interventions are designed, implemented and expected to be adopted with little insight on the culture and context in which they are to function sustainably. Culture is defined as the knowledge that people use to develop shared beliefs, practices and norms that distinguish one group of people from another, e.g., the culture across different specialties, organisations or countries can influence and shape behaviours and intervention outcomes[23]. We report on the findings of a study, which was conducted at the macro and meso level, investigating the development and implementation of ASP in different healthcare settings across five countries (England, Norway, France, India, and Burkina Faso). The countries in this study were conveniently selected to represent different healthcare systems and cultures, different resources and facing very different challenges in terms of healthcare associated infections (HCAI). Conducting this study across these different countries provided a unique opportunity to map the implementation process of ASP to generate evidence base for the most effective means of implementing sustainable and successful interventions in healthcare organisations. It is important to conduct this study across different economies, at different stages of implementation of stewardship to enable bi-directional learning. Norway, France, and England represent high-income countries in this study as defined by the Organisation for Economic Co-operation and Development, India is classified as a lower middle-income country, and Bukina Faso is classified as a least developed country[24]. This study was conducted at cross-professional, cross-specialty and cross-national level to help identify the challenges facing healthcare systems in the implementation of ASP, and the cultural determinants that shape and drive interventions. The objective of this study was to map the key contextual, including cultural, drivers of the development and implementation of ASP across different resource settings. The countries in this study were conveniently selected to represent different healthcare cultures, different resources and facing very different challenges in terms of HCAI. Conducting this study across these different countries provided a unique opportunity to map the implementation process of ASP to generate evidence base for the most effective means of implementing sustainable and successful interventions in healthcare organisations.

## Methods

### Overview of the healthcare systems in each country

Prior to selecting the countries in this study, a search of the grey literature was conducted to assess the state of the healthcare system in each of the countries. This search was undertaken to identify the existing stewardship programmes in each country, together with the key indicators in relation to antimicrobial resistance and ASP. This data helped provide contextual background knowledge for each country and helped the analysis of the qualitative data by providing background detail. We used the Centre for Disease Control (CDC) components for stewardship to assess the existing ASP activities in each country[25]. The CDC components were used because they have longest established record of producing standards for ASP and are the most widely recognised worldwide.

### Face-to-face interviews

The study interview guide was piloted in England and in India (via telephone interviews before commencing the study). Data collection from the field was carried out in Norway (May 2015), France (December 2016), England (March 2017), Burkina Faso (June 2017), and India (April 2017). The duration of data collection in each country was two weeks. The key collaborators in each country were invited to participate in this study because of their existing work in ASP. Through snowballing and purposive sampling, the key collaborators in each country nominated healthcare professionals with roles in ASP. Individuals showing an interest were contacted by the collaborators and a mutually convenient time arranged for face-to-face interviews. Informed consent from participants was obtained prior to each interview. The key themes emerging during the interviews were investigated in more depth through further questioning during the interviews. Methodological triangulation was achieved through respondent validation and reflexivity. Data triangulation was achieved through collecting the data over different periods of time, from different persons, across different hospitals, teams, and specialties. This approach of combining data from different sources, and persons helped increase the validity and reliability of the data collected, and reduced observer bias.

In Burkina Faso, AP conducted the interviews in the local language (French). The interview guide had been translated to French (English and French interview guides available as supporting materials S1 and S2). All interviews were audio recorded and sent to be transcribed verbatim by a transcription company. The transcripts from Burkina Faso were translated to English by AP. The interviews in the other countries were conducted by EC, in English.

### Ethics approval and consent to participate

Ethical approval to conduct this study was gained from all the participating countries by the country collaborators (Burkina Faso AP and XFL, England EC, France J-CL, India SS, Norway IS). The following ethical committees granted approval for this study: Comité d’Ethique de Recherche en Santé (CERS) du Burkina Faso (Burkina Faso); Imperial College Joint Research Office Ethical Board (England); Amrita Institute of Medical Sciences Ethics Committee in Ponekarra (PO), Kochi (India); Comité d’Evaluation de l’Ethique des projets de Recherche Biomédicale (CEERB) (France); The Data Protection Officer at Haukeland University Hospital, representing the Norwegian Data Protection Authority (Norway). Prior to interviews, verbal and written informed consent was obtained from all participants. The participants were emailed the study information sheet prior to the interview. On the day of the interview the interviewer verbally described the study to the participants. An informed consent sheet was signed by the participant prior to starting the interview.

### Data analysis

Familiarisation with the data began by conducting the interviews (EC, AP), listening to the recordings of the interviews and reading the transcripts (EC, Anne P, IS, BS). Nvivo 11 software (QSR International) was used for analysis of all the qualitative data from field note observations and the transcripts.

The analysis and data collection were simultaneous using established methods of grounded theory [26–28]. Analysis was through an iterative process and constant comparison using initial coding, going back the field for further data collection, and focused coding. The key emerging categories and ideas were analysed using constant comparison, and theoretical sampling was applied until the categories were saturated. The initial analysis was performed by EC. Co-authors IS, BS, Anne P, SS, and AH provided contextual and interpretive insights into the transcripts and the emerging data from each country. They each read the transcripts from their respective countries and provided analytic input into the data gathering and theoretical sampling. The cultural categories and relationships within the recognised categories were explored to develop the theoretical statements. The constant comparative method was also used for the analysis of the emerging themes[29]. This iterative and recursive process of moving between the coded data and the higher-level categories and themes, was repeated until the themes, and the relationship between the themes, reached saturation (i.e. now new themes or inter-relationships between them were identified).

## Results

### Overview of health systems of the countries included in this study

**Fig 1:**
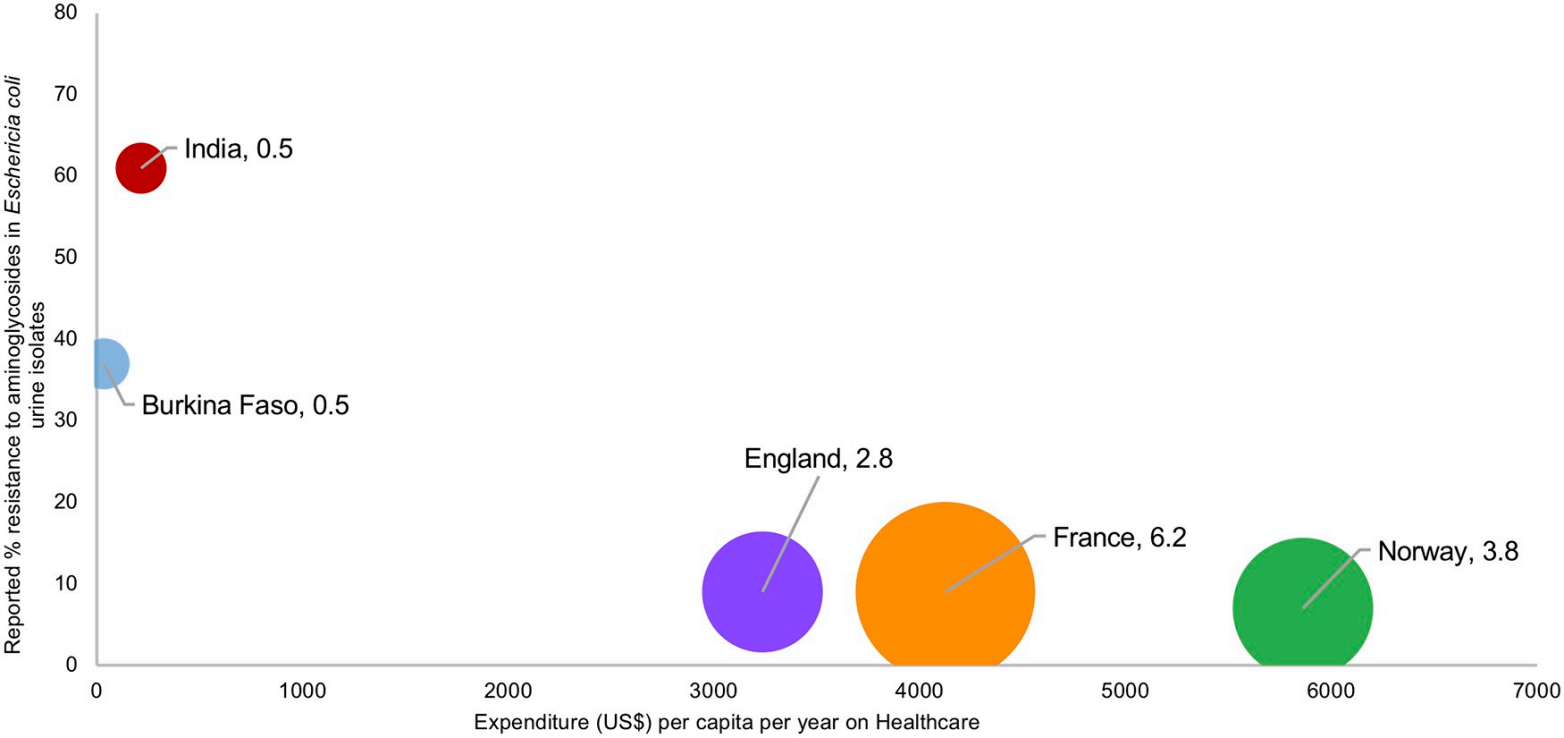
The reported % aminoglycoside resistance in *Escherichia coli* urine isolates in inpatients[1,31] against the investment in healthcare and public hospital beds per 1000 (source: The World Bank http://data.worldbank.org/indicator/SH.MED.BEDS.ZS), represented by bubble size.

The healthcare infrastructures in India and Burkina Faso are inadequate for the needs of the population, as represented by the number of hospital beds per 1000 (Fig 1). These countries also face the greatest burden of AMR, as exemplified by the reported aminoglycoside resistance rates to urine *Escherichia coli* isolates in inpatients (Fig 1). In Norway, France, and England this study found there to be country-level consistency in the provision of healthcare and the structures in place for ASP. In four of the countries, healthcare is technically free to all citizens at the point of care (Table 1). The issue of excess versus access to antimicrobials was highlighted in a recent study reporting increase in global antimicrobial consumption rates [30]. Access to antimicrobials is more challenging in countries with the highest burden of infectious diseases. The challenges of access to antimicrobials was also a finding in the qualitative study we report here. In Burkina Faso and India where there is no regulation imposed on the access to antimicrobials, patients and the public can also obtain them through informal routes.

**Table 1:** Population level data for the countries in this study.

Country data	Norway	France	England	India	Burkina Faso
**Official Development Assistance ranking**	High Income	High Income	High Income	Lower Middle Income	Least Developed
**Population**	5.2 Million	66 Million	53 Million	1.3 Billion	18.6 Million
**Doctors per 1000 population (WHO)**	4.42	3.2	2.8	0.75	0.05
**Antimicrobial consumption DDD/1000 inhabitants per day** [30]	15.4	35.4	23.2	13.5	5.8 (Figure for French West Africa, which includes Burkina Faso)
**The estimated burden of HCAI as % hospital admissions** [32,33] (India and Burkina Faso prevalence calculated by EC based on published papers [31,34–39]	5.1	6.7	7.6	25	23
**Healthcare provision**	Universal public care Federal Administration	Universal healthcare Central Administration	Universal healthcare Central Administration	Universal healthcare, access forces people into private care, Federal Administration	Universal healthcare being developed Central Administration
**Hospital accreditation**	Mandated by law	Mandated by law	Mandated by law	Voluntary	Voluntary
**Access to antimicrobials**	Only via prescription	Only via prescription	Only via prescription	No regulation easy access to black market medications	No regulation easy access to black market medications

The countries included in the study are all responding to the WHO National Action Plans for AMR, primarily via government-initiated activities. Burkina Faso is the only country which is yet to develop a National Action Plan, and currently there are no national initiatives or guidelines. Local ASP policies are based on the French system which is not fit for purpose in the context of healthcare in Burkina Faso.

This study found country level (Norway) or hospital level (England, France) antimicrobial prescribing guidelines (Table 2). The leadership commitment and appointing a single person responsible for ASP is present in England, France and Norway (Table 2). This also ensures clear lines of accountability for antimicrobial optimisation and use. In these countries there are processes in place to track the hospital level antimicrobial use and resistance patterns. In addition, these countries have implemented some form of regular reporting of antimicrobial use and resistance to healthcare professionals in hospitals.

**Table 2:** Key stewardship activities present across the hospitals in this study by country (*In India, one hospital in this study exhibited positive deviance).

The 2014 CDC Key components of stewardship	Norway	France	India*	England	Burkina Faso
Providing antimicrobial prescribing guidelines	**√** national	**√** local and national	**√** state-wide–not implementable	**√** local and national	**√** local
**Leadership Commitment:** Dedicating necessary human, financial and information technology resources.	**√**	**√**		**√**	
**Accountability:** Appointing a single leader responsible for program outcomes. Experience with successful programs show that a physician leader is effective.	**√**	**√**		**√**	
**Drug (Pharmacist) Expertise:** Appointing a single pharmacist leader responsible for working to improve antimicrobial use.				**√**	
**Action:** Implementing at least one recommended action, such as systemic evaluation of ongoing treatment need after a set period of initial treatment (i.e. “antimicrobial time out” after 48 hours).		**√**		**√**	
**Tracking:** Monitoring antimicrobial prescribing and resistance patterns.	**√**	**√**		**√**	
**Reporting:** Regular reporting information on antimicrobial use and resistance to doctors, nurses and relevant staff.	**√**	**√**		**√**	
**Education:** Educating clinicians about resistance and optimal prescribing.	**√**	**√**	**√** state level	**√**	**√** limited

Across the five countries, 61 individuals from 33 hospitals were invited to participate in the study. Nine individuals (India 2, Norway 2, France 2, England 3), refused to participate or did not respond to the two emails that were sent. In total 52 participants from 24 hospitals were interviewed (Table 3). The male to female ratio of participants was 2 (35 male and 17 female). The years in practice in the stated profession of the participants ranged from 5–42 years.

**Table 3:** Study participant data.

Country	Organisation type (study site number)	No of beds	Participant Profession
Norway	*University Hospital A	1100	Pharmacist
			Doctor
			Doctor
			Doctor
			Doctor
			Doctor
			Doctor
			Doctor
	Regional Hospital B	81	Pharmacist
	University Hospital C	947	Doctor
			Doctor
	Private Hospital D	184	Doctor
			Doctor
France	*University Hospital E	987	Doctor
			Doctor
			Doctor
	Regional University Hospital F	1500	Doctor
	Regional Hospital G	735	Doctor
	Hospital H	475	Doctor
	General Hospital I	800	Doctor
	University Hospital J	300	Doctor
	University Hospital K	1000	Doctor
India	Government University Hospital L	1250	Doctor
	Missionary (Private Charitable) Hospital M	1800	Doctor
	*Private Charitable University Hospital N	1350	Surgeon
			Pharmacist
			Pharmacist
			Doctor
			Doctor
			Surgeon
	Private Hospital O	420 (Full operational capacity 1100)	Doctor
	Government District Hospital P	750	Doctor
	Government University Hospital Q	600	Doctor
			Doctor
	Government University Hospital R	500	Doctor
England	*University Hospital S	890	Doctor
			Pharmacist
			Pharmacist
	University Hospital T	450	Doctor
			Doctor
	University Hospital U	1000	Doctor
			Pharmacist
	University Hospital V	1100	Pharmacist
			Nurse
Burkina Faso	University Hospital W	600	Pharmacist, PHD
			Pharmacist
			Doctor
			Doctor
	University Hospital X	800	Doctor
			Doctor
			Pharmacist
			Pharmacist

*Denotes the reference study site in each country.

### The key emerging themes

Analysis of the interviews, and the observational data identified key insights into the determinants of ASP in these different settings. These insights are summarised in Table 4 and are described in detail in the sections below.

**Table 4:** Key emerging themes from the data.

Emerging theme	Theme explanation	Examples	Implications
Government and state infrastructure are perceived as determinants of ASP	Government or state support and endorsement of ASP initiatives is a key determinant in implementation and adoption of ASP, however depending on the degree and level of involvement it can be perceived both as a facilitator and a barrier to effective ASP.	Government or state involvement in ASP is present in England through the Department of Health guidelines and hospital inspections. These government initiatives can be considered too invasive and disruptive to ASP. In India and Burkina Faso, there are no state involvement in ASP, and this is perceived by the participants in this study to be the reason for no impetus to implement any ASP in hospitals. Infrastructure to support ASP through legislation of access to antimicrobials and accreditation of hospitals is present in England, France and Norway, but absent from India and Burkina Faso.	In England, too much government interference in ASP through conflicting messages creates disruptions to the healthcare system in relation to ASP. Lack of infrastructure to legislate and control access to antimicrobials is a major gap in India and Burkina Faso and needs to be addressed for the widespread adoption of ASP. However, local championing and leadership can still result in successful ASP in the absence of central government endorsement.
Professional boundaries dictate involvement in ASP	Implicit and explicit professional boundaries determine the roles that healthcare professionals can adopt in ASP.	Entrenched hierarchies within organisations and between the professions define the boundaries of power and influence in ASP. It is an accepted maxim that doctors are the decision makers in hospitals in Norway, France, Burkina Faso and India. These are implicit (through widely held beliefs and cultural norms), and explicit (through legislation and job descriptions and definitions). These boundaries determine what role individuals from different professions can have in ASP.	England in this study remained the only example of country-wide acceptance and involvement of nurses and pharmacists in ASP. The examples from England and one key site in India exhibiting positive deviance, demonstrate that these healthcare professionals can have an essential role in ASP. Furthermore, the example from India, supports the theory that with local champions these healthcare professions can overcome the professional boundaries and national/organisational hierarchies and establish a role for themselves in ASP.
Social norms and values in relation to antimicrobial decision making are different in medicine versus surgery	Surgical and medical specialties are reported to exhibit different social norms, values and behaviours in relation to ASP	Across all the countries in this study the surgical specialty was identified as being more difficult to involve in ASP, and surgical teams’ antimicrobial prescribing was considered to need more attention as part of ASP.	Historically ASP have targeted medical specialties. Antimicrobial prescribing across the surgical pathway needs to be addressed as part of ASP.

#### Government and state infrastructure as determinants of ASP

In India and Burkina Faso, there is great disparity in the level of access to healthcare, and the quality of healthcare provided. There is variation in the availability and type ASP models in secondary care. Bureaucracy and inefficient systems force individuals to seek private healthcare. Government hospitals often have limited budgets for patient services. The diagnostic capability of the microbiology laboratories of the government hospitals is often technologically 10–15 years behind. In addition, due to the relaxed regulations and no mandatory accreditation for hospitals there are a variety of hospitals and healthcare providers which serve the population at different costs. Often, by the time patients are hospitalised they have already taken several different over the counter antimicrobials (ranging from a single tablet to a full course), and the lack of access to or availability of medical records makes it impossible to know the full past medical history (including infections or antimicrobial exposure) for individual patients, Table 5 (T1Q1, T1Q2). The pharmaceutical companies can provide barriers to effective ASP through the influence of their representatives on the prescribing behaviours of doctors (Table 5, T1Q3) and through the production of substandard antimicrobials (Table 5, T1Q4). Furthermore, there is the risk of black market drugs infiltrating the secondary care supply chain.

**Table 5:** The data from the interviews by theme.

Theme	Quote	
**T1** Government and state infrastructure are perceived as determinants of ASP	Q1	*‘Now in Indian scenario access to healthcare is a problem*, *very many places*, *the prescriber is alternative medicine*. *First contact is an alternate medicine*, *first contact is a quack and first contact is a nurse*, *first contact is a pharmacist*, *a trained or an untrained pharmacist*.*’* **Medical Superintendent, India Hospital N**
	Q2	*‘…people come from many … different hospitals after receiving different courses of antimicrobials also*. *So*, *it’s very difficult to grow them* [microbiological cultures] *also because it’s all pre-treated with antimicrobials*.*’* **Consultant Microbiologist, India Hospital M**
	Q3	*‘However*, *we can mention pharmaceutical representatives who have great influence on the prescription of medicines*, *particularly antimicrobials*. *There are not enough therapeutic protocols in the hospital*, *so I think that these representatives can easily influence antimicrobial prescription*.*’* **Consultant, Burkina Faso Hospital X**
	Q4	*‘When I was part of the government defender body I realised it is so difficult to work because they are a multinational with money*, *they can change the government but you cannot just keep on saying that*, *it’s so difficult it’s a mafia…have identified 24 manufacturers who are producing counterfeit drug*, *it may be small but there is an effort*.*’* **Medical Superintendent, India Hospital N**
	Q5	*‘… ultimately none of us wants to lose what for us was going to be £750*,*000 of income if we didn’t achieve the target*. *So*, *it was again a hearts and minds approach*. *It wasn’t*, *we’re going to track down every bad prescription and tell people off and report them to the chief executive*. *It was*, *we’re going to go out and present people the facts and the information*, *make a compelling argument*, *and appeal to their good nature to cooperate and be part of this push all together*. *And that worked*, *it seems to have worked*. *So that we’ve been monitoring the trends and we’re*, *on track to meet the CQUIN*.*’* **Consultant, England Hospital U**
	Q6	*‘I fear*, *personally*, *I think it’s negatively impacted the pharmacist’s job role within the antimicrobial team*. *Our role pretty much now is to drive the CQUIN agenda and make sure that we’re meeting all the targets*, *so we really have to pull back from what I think is an important day to day role so attending ward rounds*, *and we just don’t have time to do that anymore*, *we’re purely office based trying to hit the CQUIN targets*.*’* **Pharmacist, Hospital S**
	Q7	*…*..*people* [referring to the body responsible for CQUIN targets]*… feel that they have an informed opinion or an opinion which is worthwhile listening to and acting on*, *which you wouldn’t ask a surgeon*, *a heart surgeon*, *you wouldn’t say to a heart surgeon well I think you should be doing this sort of clinical practice…*. *that sort of CQUIN would come from within the specialty itself…*. *as an illustration*, *David Cameron saying we’re going to reduce our inappropriate antimicrobial prescribing by 50% by 2020*. *How do you know what’s inappropriate*? *It’s all very well*, *it all sounds really excellent stuff*, *but it opens up so many questions that are impossible to answer*. *I was down the Department of Health and we were looking at how we’re going to measure inappropriate prescribing … and the universal answer was well we can’t*, *we don’t know*, *how would you know whether it’s inappropriate or not*?*’* **Consultant, Hospital S**
	Q8	*‘To set up a guideline*, *anyone can do it*. *What I would suggest is everyone come up with antibiograms and make it*, *mandatory is the word I think I can use*, *make it mandatory that*, *see*, *we have come up with data from South India*, *this is from North India*, *East and West*, *whatever*, *and study that in detail and the government itself should say*, *you have to implement this or your job is in jeopardy*. *Something will happen…*.*my issue is that we all get our salary*. *We get our salary no matter what happens*, *and I’m afraid that doesn’t happen*. *Once you don’t do your job properly*, *you’re sacked*. *Here you are protected by the government itself because there is no point in taking the mike and talking about stewardship and government policies*. *You have to implement it*, *thinking that their job will go if they are not going to follow this*.*’* **Professor of Microbiology, India Hospital Q**
	Q9	*‘…here we don’t have an active role* [in ASP]. *We don’t see the patients and the clinicians have no direct communication with us*. *But*, *nowadays*, *this antimicrobial stewardship and all are gaining importance so*, *at government level itself*, *certain initiatives are being taken under Dr X* [Medical Superintendent at Hospital N]. *So*, *this*, *even recently a meeting was convened at the capital of Kerala where all the head … of medical colleges … and they have met*, *I hope they have started discussing what could be done in view of this*.*’* **Consultant Microbiologist, India Hospital R**
	Q10	*‘Also of course*, *there is the … structure on the Norwegian healthcare system with the Department of Health as the owner of the regional health authorities*, *and the Department of Health sends an order every year to the regional health authorities*, *this could contain both*, *they could demand an antimicrobial stewardship programme should be implemented in hospitals*, *but also this could come from the regional health authorities*, *and out to the other health trusts and hospitals*. *So that is probably the most effective way of getting antimicrobial stewardship programmes in Norwegian hospitals*.*’* **Consultant Infectious Diseases, Norway Hospital A**
	Q11	*‘There is antimicrobial stewardship*. *It doesn’t matter who is involved but there is a stewardship programme and there is willingness to make it better*. *In the small hospitals*, *I mean less than 300 beds*, *or private hospitals*, *it’s not done because there are no specific practitioners there is no time to do it*, *there is no money*. *The reason why it is moving now is the fact that our healthcare indicators now has one dedicated to antimicrobial stewardship*.*’* **Consultant, France Hospital F**
	Q12	*‘In Norway it’s hospital infections specialists who have that role in many hospitals but it’s very difficult to have a*, *to get doctors into that speciality*. *It’s very difficult*. *So in this hospital there are no full time hospital infection control specialists*. *I have a job and infectious disease specialists have a job*, *part time*, *not full time*.*’* **Head of Microbiology, Norway Hospital C**
**T2** Professional boundaries decide involvement in ASP	Q13	*‘In France a lot of advice is obtained from ICU* [Intensive Care Units] *doctors*, *sometimes in small hospitals ICU physicians serve as a reference for antimicrobial advice…*. *I think that physicians in the hospital are more confident in the advice given by an ID physician compared with advice given by a pharmacist or microbiologist*.*’* **Consultant, France Hospital E**
	Q14	*Interviewer*: *‘What do you think is the biggest barrier to changing people’s behaviours*? *From your experience*?*’* *Participant*: *‘Hierarchy*. *I think we need to accept that doctors are not some sort of gods*. *It’s like an occupation*. *Doctors are doctors*. *It’s only an occupation*. *Pharmacy too*, *everyone is important in this world*.*’* **Consultant, France Hospital F**
	Q15	*‘…it’s sort of ‘are you a member of the family or no*? *And this is why it’s impossible for nurses to be involved*. *‘She’s a nurse*, *she’s only a nurse’… and about pharmacist it’s also the same*. *When you speak to pharmacists about being involved some of the pharmacists they don’t want to be involved in the programme*. *They say “I am not a clinical doctor*, *I don’t know …do you imagine that as a pharmacist I am will call a doctor and tell them you have made a bad choice*? *It’s impossible*!*”‘* **Consultant, France Hospital E**
	Q16	*‘But there is …*. *a north south divide*, *so the hierarchy in the north is amazing*. *The nurse will not be able to open her mouth and say*, *this is illegal*, *like in England* [the participant had spent time training in England], *the nurse will just rap my knuckles and say that*, *you’re doing this wrong*. *They won’t have the guts to say that in the north*. *Kerala*, *because the communist culture*, *it’s a lot better*, *because they have*, *you can’t scream at them the way they do in the north*, *it’s a very bad*, *very*, *very bad system*.*’* **Surgeon, India Hospital N**
	Q17	*‘The* [senior microbiologist] *has always taught us*, *and it’s an approach that I've adopted*, *is that as microbiologists*, *and even as ID physician*, *especially here and other places where we have a consult service and we don’t have our own patients*, *we’re guests*, *and in the same way that you would expect a guest to come to your house and behave a certain way… you have to do the same thing …*. *so you go to the MDT* [multidisciplinary team meeting] *and you kind of establish a rapport … and then you rely on making an impression but also gaining their trust for them to invite you back*. *You* can’t go along to the first meeting and tell them they’re doing everything wrong and override all their decisions.’ **Consultant Microbiologist, England Hospital T**
	Q18	*‘If they want*, *we go*. *And if they want me to come for rounds*, *I go*. *It’s their decision*. *I don’t go actively from my side*, *because that will*, *that doesn’t work*. *They are in a different place*, *the whole hospital they are going around*. *They have other issues*, *they have procedures*. *So our timing’s … So if they want something to be done and they call me*, *I go*, *have a look at it*, *give my advice*.*’* **Consultant Microbiologist, India Hospital N**
	Q19	*‘Here*, *we do not directly communicate*, *we send the labs to the central collection area and from there the clinicians collect*, *but in certain situations*, *when we think we really need to alert them*, *we call them directly*. *I’m not fully satisfied with the response*. *I think here the communication is a little bit low between the clinical side and the lab side and what I feel is*, *if the communication is better*, *there can be a significant change*.*’* **Consultant Microbiologist, India Hospital R**
	Q20	*‘So what I’ve understood from this about nine years of doing this…*.. *you need a strong support or commitment from the top administration*. *And when I say support*, *it should be visible meaning it is not that the CEO tells you in a closed door meeting …*.. *What I mean is that in the mortality morbidity meeting*, *in the CMEs* [continuing medical education] *of doctors*, *when you have the whole faculty meeting*, *at that time when the boss says that he’s behind you*, *supporting you for this*, *that is visible support*. *So we need support which is visible*, *like the top management talking to other people that*, *no*, *I am with this team*, *not just between ourselves saying that I am with you*. *So that is one thing which I noted which makes a difference*. **Consultant, India Hospital O**
	Q21	*‘The best in France is here*. *I was convinced when starting that to make a good job we had to work closely with pharmacists and microbiologists… So one of my main drivers was always to be with these collaborations*. *And now infection control*, *and it works well*. *I think it’s quite exemplary*. *I’m quite proud of that*. *Of the excellence of the relationships between all these partners*. *And I think it is particular to the team here*…*’* ***Consultant*, *France Hospital E***
**T3** Social norms and values in relation to antimicrobial decision making are determined at specialty level	Q22	*‘Generally ICU’s are better at using the guidelines than other wards*, *and on other wards*, *specialties are better than general doctors and medicine is better than surgery*.*’* **Consultant, France Hospital F**
	Q23	*‘We are doing some audits on surgical wards around behaviour*. *How they close and open wounds during the surgery… and the heads of surgery had the wedding rings and performed the surgery wearing the rings…*. *we fed back to them and to the head of nursing*, *saying “thank you for this*, *but we found a little problem*. *Doctors and nurses should have their hand free*.*” And within five minutes of sending this email*. *The head of surgery answered*: *‘Doctors will never take their ring off…”* **Consultant, France Hospital F**
	Q24	*‘Like it is generally said that there is a cardiovascular thoracic surgeon*, *there is cardiologist*, *there is god in hierarchy and then there are physicians and surgeons*. *So these group of people they don’t talk*, *they don’t allow you to talk also because they do what they want to do because …*. *transplant surgeons and cardiovascular thoracic they don’t listen*. *But fortunately they have seen that our intent is ethical and intent is quality and we are not driving any programme on commercial interest…’* **Medical Superintendent, India Hospital N**
	Q25	*‘the problem is that usually*, *they trust us to treat the infection but the infection also needs medical and surgical management and sometimes we are unable to change the surgical procedures for the best infection management outcomes*. *So*, *sometimes it’s difficult even if they leave us to treat infection the discussion of the surgical procedure and management is difficult to obtain*. *Sometimes the department gives us the trust but it is relative trust*. *This is very technical and strategic work and it is political and it’s about relationships*, *confidence etc*. *It takes time*, *years to build*. *Some departments are really resistant…’* **Consultant, France Hospital E**
	Q26	*‘I find most surgeons feel it is my patient which is at stake so I must treat him irrespective of cost or whatever stakes that are there*, *I really don’t care about tomorrow*. *It’s just like global warming isn’t it*? *Like let me use it now*, *I don’t know if this is going to come a million years later*, *we don’t realise that some of those effects are for our children*. *In antimicrobials I feel it’s in our very own patient as well*.*’* **Surgeon, India Hospital N**
	Q27	*‘The surgeons of course*, *they are not so much interested in antimicrobials*. *Internal medicine*, *of course they are*. *They are*, *infection disease specialists are a team in our subspecialty internal medicine*, *so they will get information from the infectious disease specialists more than other specialties in the hospital*.*’* **Head of Department, Norway Hospital C**
	Q28	*‘And if you compare Norway to the UK which*, *I think we are more I guess egalitarian but …*. *it’s not accepted that someone has his own way of giving antimicrobials*, *although you are German and*, *or French and come to work here*, *you have to adjust*, *and if you don’t adjust the medical*, *the chief of the medical doctors would say no*, *and you have to adjust or I would do it*, *I don’t have to do that because it’s*, *the leadership in the medical department is quite strong*. *if you look at*, *different surgeons can often do things a little bit different but we work to standardise everything … and all the doctors do everything with the same procedure*, *and we want that there*. *Chief surgeon of gastro surgery decides how we do it in this hospital*, *and it’s all up to four different doctors to treat the patient differently*. *That*, *we have some work to do there but when it*, *in regards antimicrobials*, *or using a ring*, *or stuff like that*, *we do have authority … to change that behaviour*. ***Medical Director*, *Norway Hospital D***
	Q29	*‘We’ve got one of our trauma and orthopaedic consultants who actually goes around surgical wards with one of our pharmacists and audits other surgeon specialties and goes and tells them their surgical prophylaxis is too long*. *And that again…*. *is just much more powerful than me going with a big report and saying*, *we snooped behind your back and did this audit and this is…’* **Consultant Pharmacist, England Hospital V**

‘*The regulation is very poor*. *Companies making generic antimicrobials make two formulations*, *one for export*, *one for use in India*, *in one study on cephalosporins*, *the Indian generic version had 15% less potency*. *We had a case of aspergillosis*. *The drug of choice is voriconazole*. *In the Indian market*, *there are voriconazole from 200 rupees to 2*,*000 rupees*. *We gave the patient the option*. *He chose the cheapest one*, *the Indian generic one*. *We gave the correct dose of voriconazole*, *the infection did not respond*. *Then we did the therapeutic drug monitoring*, *we found that the concentration was at least five to six time less than the original drug … so we had to give five to six times the dose of the generic drug*.*’* Medical Microbiologist, India Hospital N

In the UK, resource is limited in a different way. The National Health Service (NHS) is under pressure to save money year on year, both in staff resourcing and in consumables such as drug expenditures. At the same time the healthcare system is subject to regular disruptions in the form of restructuring of the workforce and management teams. During this study, NHS England launched the Commissioning for Quality and Innovation (CQUIN) initiative for reducing the burden of AMR and sepsis[40]. In secondary care CQUIN provides financial incentives to hospitals for gathering and sharing data on antimicrobial consumption and review, and a further incentive for demonstrating reduction in antimicrobial use for specific agents. The participants from English hospitals included in this study had mixed responses to the impact of the CQUIN. Some considered it as an opportunity to get the rest of the organisation to focus on ASP (Table 5, T1Q5). Others, though they had participated in the initiative perceived it to be more disruptive and lacking insight into the practicalities of ASP (Table 5, T1Q6 –Q7).

The English experience is at odds with the India and Burkina Faso experience. In these countries, central government involvement is lacking but is attributed a high value by healthcare professionals, whereas in the UK it is attributed a low value by healthcare professionals, though it is an active driver of ASP. In India and Burkina Faso healthcare professionals consider ASP to be the responsibility of central government. Unless driven by central government and mandated by law, ASP initiatives will not take root in hospitals (Table 5, T1 Q8 –T1 Q9). In France and Norway central government led health indicators are driving the need to develop some level of ASP in hospitals. In Norway, a country beginning to initiate ASP, government input is perceived as a facilitator to bring about change in ASP (Table 5, T1 Q10). Limited resources at hospital level can hinder the implementation of ASP. In smaller rural hospitals lack of recruitment of staff and access to expertise pose obstacles to the implementation of ASP (Table 5, T1Q11 –Q12).

#### Professional boundaries decide involvement in ASP

In many parts of the world, including France, Norway and India ASP is still predominantly led by doctors (including infectious disease doctors or medical microbiologists). The role of the wider healthcare workforce in ASP is undefined and limited. France has a long history of ASP in its secondary healthcare. Traditionally the treatment of infections has been the remit of intensive care physicians and the intensive care units still play a pivotal role in ASP, and in infection management of patients (Table 5, T2 Q13). This has led to barriers to the ASP team to be accepted as a valid source of advice in the ICU setting, and the doctors working in ASP teams can be held at arm’s length by clinicians and regarded as a ‘we will call you if we need you’ service rather than a more collaborative service. This type of professional boundaries still abounds in the clinical setting and delineates who can do what. In the countries in this study, bar England and Burkina Faso, pharmacists do not traditionally have an active role in ASP. Traditional professional hierarchies remain entrenched in healthcare systems, leaving the nurses and pharmacists with little expectation to be part of the antimicrobial decision making (Table 5, T2Q14 –Q16). Within the medical profession there remain also concerns of stepping into another specialty’s territory. The ASP teams report an acute awareness of the sensitivity of dispensing advice to other clinicians about the care of their patients (Table 5, T2 Q17 –Q19). To be accepted by the other clinical professions, the participants from Indian hospitals expressed a greater need for support from the hierarchy in the organization (Table 5, Q20). Despite the country level propensity for ASP to be exclusive to doctors, the key study sites in each country exhibited similarities in the level of openness and inclusion in ASP with emphasis on and appreciation of interdisciplinary collaboration (Table 5, T2Q21).

#### Social norms and values in relation to antimicrobial decision making are determined at specialty level

The study participants reported an awareness of the different practices in relation to antimicrobial prescribing amongst different specialties. One finding across the countries was the challenges facing ASP teams in engaging with and optimising antimicrobial use across surgical teams. Generally, it was reported that surgical specialties are the least likely to adhere to antimicrobial prescribing guidelines, whereas ICU teams being protocol driven and used to multidisciplinary input are the most likely to adhere to them (Table 5, Q22 –Q23).

France and India both present the more hierarchical cultures[41] and this was reflected in the findings with the surgeons reported as being on top of the hierarchy in hospitals. The surgeons’ position in the hierarchy manifests itself as a reluctance to follow general expectations of behaviour in relation to infection management and antimicrobial prescribing. Due to the lack of attention to the medical care of the surgical patients and ineffective co-management, the medical needs of surgical patients can be neglected and overlooked (Table 5, Q24 –Q25). The reasons for these reported differences in antimicrobial decision making and infection management amongst the surgical teams is perceived to be that the priority of surgical teams is not optimisation of antimicrobial decision making, rather it is infection avoidance (Table 5, Q26 –Q27). In Norway, representing the most egalitarian culture in this study, though antimicrobial use in surgery is a recognised problem, through the hospital administration, efforts are made to standardise clinical care across the specialties, including areas considered to be a priority for the organisation such as antimicrobial prescribing (Table 5, Q28). The overall impression of the Norwegian healthcare system is one where hierarchies do not exist in the same way that they exist in the other healthcare systems in this study. For example, visual hierarchies are flattened by the fact that all hospital staff from the managers and hospital chiefs to the cleaners are required to wear the same white uniform when in clinical areas.

In England, one hospital’s solution to engaging with surgeons is through multidisciplinary collaboration and inclusion of surgeons in the ASP initiatives. The surgeons, it is believed, will be more receptive to the ASP message, if it is delivered to them by a peer, i.e. someone they consider to be from their own discipline (Table 5, Q29).

#### A case study of success in implementing ASP in India

**Fig 2:**
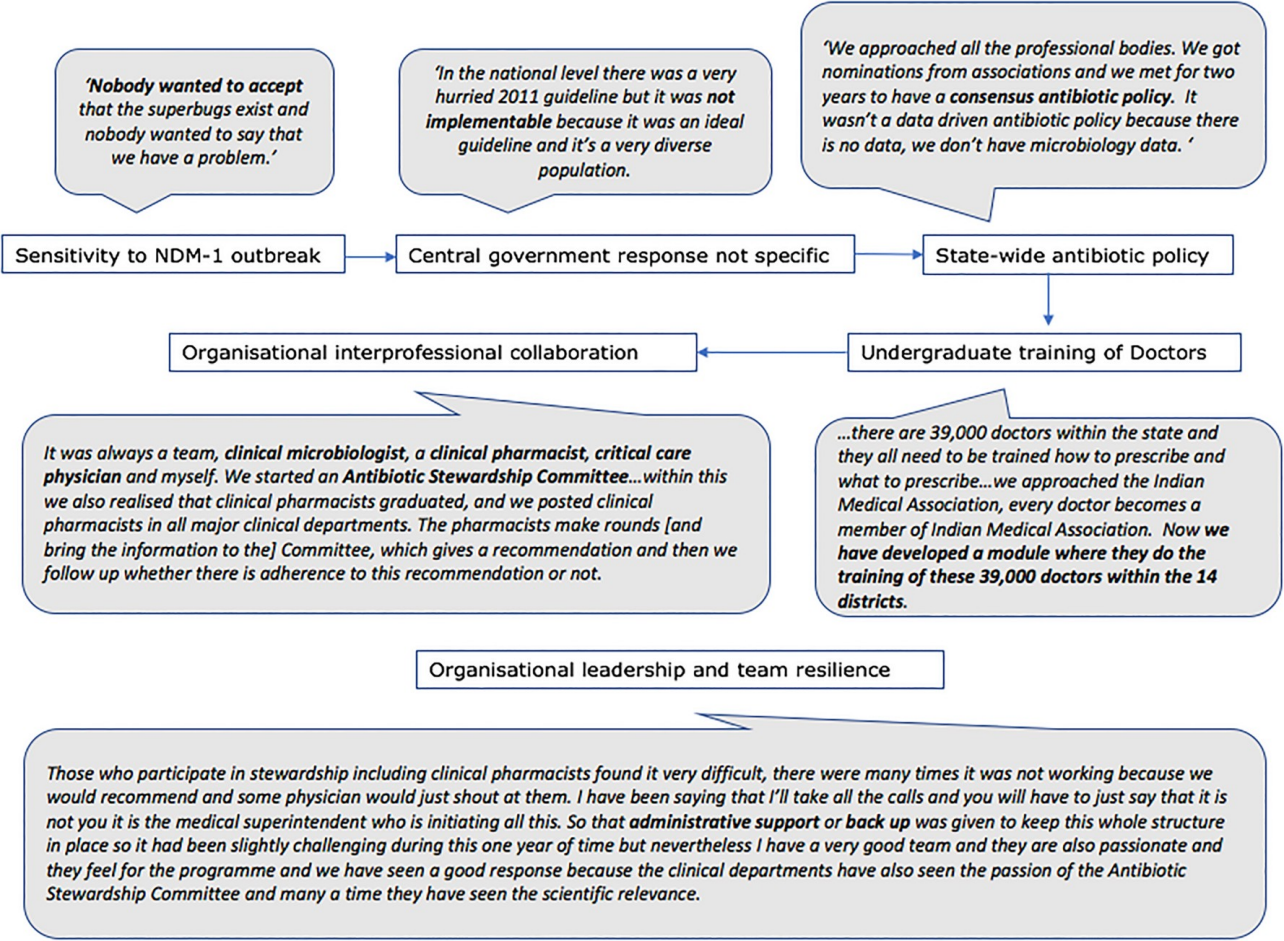
Development of the ASP at hospital N as recounted by the ASP staff.

In India one hospital stood out as a case study of positive deviance and success in implementing ASP. The process of developing ASP at Hospital N, as recounted by study participants, is summarised in Fig 2. Hospital N is a tertiary referral care charitable not-for profit organisation founded in 1997 in the state of Kerala. The key features of this ASP are listed in Table 6. Realising that national programmes were not implementable locally due to lack of contextual insight, the staff at the hospital decided to tackle the issue at state level. By approaching the Ministry of Health in the state and the medical colleges, they were able through consensus to develop a state-wide antimicrobial prescribing policy and an education and training programme for undergraduate medical trainees. Organisational champion and leadership, together with team resilience resulted in successful implementation of an ASP, in country that remains hierarchical and lacks national ASP policies.

**Table 6:** Key components of the ASP at hospital N.

Components of Stewardship Programme	Key functions	Components
A hospital wide antimicrobial policy and antibiogram smartphone application	Policy accessible online App on smartphones	The key tools of ASP implemented hospital wide There is a mechanism for escalating recommendations through the hierarchy Key recognised ASP activities adopted as social norms The physical environment adapted to accommodate ASP e.g. electronic records
Reserved antimicrobial list– 13 antibiotics and antifungals on the list	Pharmacist review every patient on these agents, they feedback to the prescribers recommendations for review or de-escalation. This information is discussed daily in Stewardship Committee	
Antimicrobial recommendation form	Completed by pharmacists reviewing the patients on antimicrobials with a recommendation to the medical team and placed in patient notes	
Electronic health records (Developed in-house)	Supports the team in identifying patients on the reserved antibiotics and to produce consumption reports	
Interdisciplinary Antimicrobial Stewardship Committee—including surgeons, pharmacists, and Infection Prevention and Control (IP&C)	To meet daily to review and discuss patients Any difficult cases referred to the medical microbiologist and medical superintendent by the pharmacists	The social environment supports a collectivist approach with values placed on interdisciplinary collaboration
Surgical surveillance	Surveillance of surgical site infection and the introduction of a Standard Operating Procedure for IP&C and antibiotic use in cardiothoracic surgery	Value placed on interdisciplinary collaboration
Organisational leadership	The programme driven by the medical superintendent ensures continuity, acceptance, and leadership	Leadership support helps ensure desired ASP activities are entrenched as social norms over time

## Discussion

This study set out to investigate how antimicrobial stewardship is developed and implemented in different healthcare settings and economies. The findings highlight the variation in healthcare and population needs, investment, and contextual and societal norms between these countries. The threat of AMR and HCAI however, and the need to react to these threats is faced by all healthcare systems, though at different levels of severity. Healthcare professionals reported facing similar challenges in the design, development, and implementation of ASP across contextually different healthcare economies and structures. Resources were considered a limitation in development of ASP across all the healthcare economies, despite the variance in per capita expenditure in healthcare, and healthcare workforce capacity. In India and Burkina Faso, the infrastructure and expenditure in healthcare is underdeveloped, and the healthcare workforce inadequate to meet the needs, whilst the demand for healthcare is highest. The lack of infrastructure, poor regulations relating to access to antimicrobials, and the voluntary accreditation of hospitals prohibit the standardisation of care that is reported in the other three countries. The access to antimicrobials remains a critical challenge in India and Burkina Faso, exposing the risk of black market and less potent antimicrobials infiltrating the supply chains across the healthcare system. In addition, lack of regulation means that the patient and the public have many informal routes to obtaining antimicrobials. To optimise antimicrobial use and establish effective ASP in such settings, first there needs to be equitable access to standardised antimicrobials across the entire healthcare pathway. This raises the question of the ethics of restricting excess antimicrobial use through ASP, in settings where inadequate access to healthcare remains a key issue. There must be a balance between reducing excess antimicrobial use without impeding access to them. ASP is essentially about optimising antibiotic use, and it is therefore critical that it should not impede access in vulnerable populations in resource limited settings.

Resource restrictions to implement ASP apply in all countries in this study. In Norway, a geographically broad country, with a dispersed population in rural areas, access to microbiological laboratories [9,42] has already been reported as a limiting factor in implementing ASP. Furthermore, as reported in this study in the larger hospitals infectious diseases and IP&C posts remain vacant. India faces the same problem, in that infectious diseases is not a recognized specialty and therefore there is a gap in specialism and expertise, which is reflected in the strains on ASP. In England and France, the healthcare expenditure and investment by the government is accompanied by externally driven targets and performance measures which can put a strain on diminishing resources. The case study from India, provides learning for other settings whereby locally led ASP initiatives that use interdisciplinary approach can overcome some of the resource limitations.

Policies and guidelines, the bedrock of ASP, are not universally implemented, and where they are implemented the value attributed to them varies. In Burkina Faso and India, where guidelines are much needed, the national guidelines that were implemented were not fit for purpose or context. In England and France, the two countries with the longest established ASP, the stewardship agenda has moved beyond policy and guideline provision. It is almost as if these interventions have been saturated. The ASP teams and the Department of Health and national bodies are striving to convince their clinical peers in the hospital setting to adhere to the guidelines, and to convince them of the necessity to comply with policy recommendations. In India and Burkina Faso, the overwhelming reliance on the state endorsement has meant that polices and guidelines are likely to be adhered to if they are ratified and endorsed by the government or come with a punitive measure. In countries with little government led ASP, there is a sense that only the government can incentivise and mobilise ASP efforts. This expectation in LMICs for the government to ‘solve’ the issue of inappropriate antimicrobial use is in contrast with the perception in England that government involvement may have unintended consequences for local ASP. Arguably the level and extent of state or government input into ASP can alter the culture of practice locally in hospitals, by shifting priorities, and over time influencing values and norms. The extent of the unintended consequences of central government involvement (for example the CQUIN targets in the England) in ASP have not been fully investigated. Whilst a degree of state (top down) reinforcement and support is essential, e.g. to ensure an adequate healthcare service accessible by all, too much intervention and micro-management can have negative and disruptive effects.

This study found that despite the expectation of multidisciplinary collaboration in ASP from WHO and other international bodies, it is only still practiced in a limited number of countries. In the context of this work, across India, France, and Norway doctors remain the sole profession involved in ASP efforts. The reasons for this are manifold. The entrenched hierarchies within the medical profession [5] that impact antimicrobial prescribing has been described as part of previous research. However, it was interesting to find that the key study site in these three countries exhibited a similar culture to one another, and different from other hospitals in their own countries. This may be due to these sites being national centres of excellence with a reputation for innovation and leading the ASP agenda. Their success is indicative that with the right leadership and drive, cultural norms can be challenged and changed over time e.g. the cultural norm of the medical profession leading quality improvement interventions, such as ASP. This is more likely to be achieved in organisations that exhibit a more collectivist approach to team work and decision making as exemplified in the case study from India. Against a highly hierarchical culture, with very limited resources in the healthcare system, an increasing threat of AMR, and little government input, one hospital in India has managed to establish and sustain an interdisciplinary ASP. In part this was achieved through dedicated leadership championing the cause of ASP, and a recognition that the wider healthcare workforce including pharmacists can make a valuable contribution to clinical decision making and patient care. This example of positive deviance carries a powerful message that with the right leadership and interdisciplinary approach organisational cultures can be different to the national culture.

This study reports that surgery specialties remain universally difficult to engage with regarding ASP. The lack of adherence to ASP principles by the surgical teams also affects the medical care of the patients. Historically ASP efforts have mainly targeted medical specialties[17]. This is a critical oversight, and optimising antimicrobial prescribing before, during, and after surgery should be a central tenet of tackling AMR [43]. Most of the research in ASP in surgery is focused on antibiotic prophylaxis, and prevention of surgical site infections [17]. It is imperative however to engage more with surgical teams and try and address antimicrobial prescribing more comprehensively along the entire surgical pathway. It has previously been reported[5,11,44] that peer endorsement is an important factor in the uptake of sustainable interventions in healthcare. ASP efforts place expectations on autonomous clinicians, including surgeons to change their behaviours from what to them is a norm within their specialties and areas of practice. Often the expectation yields no direct benefit to the patient at hand but is in view of a greater good of averting the emergence of AMR and preventing HCAI. To this end, busy, pressurized surgeons working with limited resources may not immediately see the merits of ASP or prioritise related behaviours. The use of local champions and organisational leaders are important determinants in shifting antimicrobial prescribing behaviours, social norms and values over time.

### Potential opportunities to affect change

The key learning from the findings in this paper are focused on workforce development, identifying key champions to drive stewardship locally and to engage with governments and policy makers to ensure effective support for and scale up of interventions. Research on the implementation of ASP from low resource settings remains critical. By focusing within LMICs and addressing these questions in the least resourced settings, we will maximise the opportunities for bi-directional learning and the potential for scale up to any resource setting. There is a much wider workforce, including pharmacists and nurses, within healthcare that can take forward stewardship interventions in different settings.

### Limitations

There are several limitations to the methods, and interpretation of this study. First and foremost, In India, France and Norway access to the pool of potential participants was gained via key informants who were national leaders in ASP in their own countries. This may have introduced selection bias. The fact that they were identified by the key informants was intentional as the aim was to include individuals who had a role in ASP. There may have been bias in the participants feeling obliged, coerced, or under undue influence to participate due to the key informants having invited them. Having spent time in each of the lead institutions however, and observing how the teams operate, coercion is not a plausible bias in this study. One of the most advanced examples of interdisciplinary teamwork in this study is from India, a country historically considered to be extremely patriarchal and hierarchical. Another cause for selection bias and limitation in this study was language. The need to speak English (France in Burkina Faso) may have been a selection bias for the participants. In Norway and India, all university graduates are fluent in English, therefore language was not a bias for selection.

## Conclusion

At the macro level government and state infrastructures determine ASP. There is a recognised need for legislation and investment in resources to support ASP locally, however too much government involvement can lead to disruptions in ASP and redirection of limited resources. A culture of hierarchies dominates the effectiveness and reach of ASP, with professional boundaries limiting the involvement of nursing and pharmacy staff and doctors remain the key stakeholders. ASP champions and local leadership can be used to overcome hierarchical and rigid national and organisational cultures. There are opportunities for shared learning from the interdisciplinary model adopted by key study hospital in India, which could break through entrenched hierarchies. Across all the countries in this study the surgical specialties were identified as being the least likely specialty in acute care to adhere to ASP. To tackle AMR, the surgical specialties in secondary care need to be included in ASP initiatives. It is critical to develop contextually driven ASP targeting the surgical pathway in different resource settings. There needs to be a complete and transparent, shared understanding of the principles of optimised antimicrobial prescribing across the entire healthcare work force in acute care, including across surgical teams.

## Supporting information

S1 File Interview guide in English. (PDF)

S2 File Interview guide in French. (DOCX)
