# Supplementary material for: Investigating the cultural and contextual determinants of antimicrobial stewardship programmes across low-, middle- and high-income countries—A qualitative study
Source: PLoS One. 2019 Jan 16;14(1):e0209847. doi: 10.1371/journal.pone.0209847 (PMC6335060; doi:10.1371/journal.pone.0209847)
Supplement: S1 File — (PDF) [file pone.0209847.s001.pdf]

Study II: **Shared healthcare professional engagement in antimicrobial stewardship programmes – what we can learn from other healthcare systems**

APPENDIX C: Assessment toolkit for antimicrobial stewardship programmes in hospitals

Study Title: How is antimicrobial stewardship (AMS) defined and implemented across a multisite healthcare setting?

Semi-structured interview guide

Organisational level:

Roles and responsibilities

1. Who is **responsible** for antibiotic prescribing in your organisation?
    a. How is it delegated?
    b. Does it work in practice?
2. Do you think you have a role or responsibility in antibiotic prescribing in your organisation?
3. What are the **external (if any) influences such as policy, governance, inspections** for an antibiotic prescribing programme at the hospital?
4. **What or who influences** how you rank your priorities?
    a. **What influences how you allocate the available resources both human and economic?**
5. Are there any **policies or guidelines** in your organisation for antibiotic prescribing?
6. What groups of healthcare professionals are identified as having a **role in antibiotic prescribing/infection control** activities at organisational level?

Reporting structures and data

Organisational:

7. Is there a **reporting structure** for antibiotic use and prescribing in your organisation?
    a. What about national level?
8. What are the **quality indicators**, if any, for antibiotic prescribing at board level?
9. **Who** measures these?
10. How are indicators **reported**? How often?
11. How much of what is presented at the board is shared with the **average employee** in the organisation?
    a. If it is shared, how is it shared?
    b. Via what mechanism? Mass email? Newsletter?
    c. How often is it shared? Weekly, monthly, etc
    d. Is there any **demand for employees** for information/data on antibiotic prescribing?
12. Are there specific antibiotic prescribing or infection control related **committees/meetings/units**?
    a. Who is represented here?
    b. How often do they meet?
    c. Is there cross-representation at other units/teams? i.e. how is information shared across teams/committees?

Local:

13. Is there regular **local (meaning, department/unit) measuring of antibiotic prescribing**?
    a. If yes, what is measured?
    b. Who measures the data?
    c. Who is the data feedback to?
    d. How often?
14. Is any **public/patient engagement** in antimicrobial prescribing undertaken?
15. In relation to antibiotics **what kind of data** would you like to have available at organisational or local level?
    a. Why this particular data?

Individual interventions:

Participants will be asked to think of a recent intervention they implemented as part of an antibiotic prescribing programme and asked the following:

Intervention aims and measures

1. Are you aware of any recent interventions targeting antibiotic use in your hospital?
    a. What about other hospitals?
2. In YES, ask questions below, if **NO**: **are there any antibiotic related interventions, which you think should be implemented in your organisation? IF YES ADAPT BELOW TO FUTURE TENSE**
3. What was the trigger for the intervention? Outbreak? Cost-saving? AMR?
4. Why would this intervention be an improvement?
5. What were the intervention aims?
    a. Who was involved in setting the aims?
    b. Were potential unintended outcomes addressed?
6. How and what did you measure?
    a. Who collected these measures?
    b. Could existing data from the hospital pharmacy?

Feedback loop

7. Was there a feedback plan?
    a. Who received feedback,
    b. How often?
    c. In what format?
    d. Was there a structured dialogue? For example discussion or explanation around the findings?
8. Was there follow up on the feedback?
    a. If yes, please describe it

At the end:

How do you think UK is doing compared to other similar healthcare systems in relation to AMR/HCAI?

In an ideal world what would you like to see implemented on antibiotic prescribing?

What are the barriers to implement this?
