# Supplementary material for: Investigating the cultural and contextual determinants of antimicrobial stewardship programmes across low-, middle- and high-income countries—A qualitative study
Source: PLoS One. 2019 Jan 16;14(1):e0209847. doi: 10.1371/journal.pone.0209847 (PMC6335060; doi:10.1371/journal.pone.0209847)
Supplement: S2 File — (DOCX) [file pone.0209847.s002.docx]

**Annexe C: Guide d'entretien semi-structuré**

1. **Niveau organisationnel:**

Rôles et responsabilités

1. Qui est responsable de la prescription d'antibiotiques dans votre *institution*?

a. Comment cette prescription est-elle déléguée?

b. Est-ce que cela fonctionne dans la pratique?

2. Pensez-vous que vous avez un rôle ou une responsabilité dans la prescription d'antibiotiques dans votre *institution*?

3. Quelles sont les influences externes (le cas échéant) telles que le système de santé, l’administration hospitalière ou de supervision des programmes de prescription d’antibiotiques à l'hôpital?

4. Quels sont les personnes ou les paramètres qui vont influencer la façon de hiérarchiser vos priorités en termes de programme de prescription antibiotique ?

a. Qu'est-ce qui influence la façon dont vous utiliser les ressources disponibles à la fois sur le plan humain et économique ?

5. Existe-t-il des politiques ou des recommandations dans votre structure pour la prescription d'antibiotiques?

6. Quels groupes de professionnels de la santé sont identifiés comme ayant un rôle dans les activités de prescription d’antibiotiques / contrôle des infections à l'échelle de votre structure?

*Structure hiérarchique et données de votre institution*

**Organisation:**

7. Existe-t-il une *Structure hiérarchique* affectée à *la* prescription et l'utilisation d'antibiotiques dans votre *institution*?

a. Qu'en est-il du niveau national?

8. Quels sont les indicateurs de qualité, le cas échéant, pour la prescription d'antibiotiques au niveau de la *Structure hiérarchique*?

9. Qui les mesure?

10. Comment les indicateurs sont-ils rapportés? À quelle fréquence?

11. Combien de ce qui est présenté à la *Structure hiérarchique* est partagé avec l’ensemble du personnel de la structure?

a. Si elle est partagée, comment est-elle partagée?

b. Par quel mécanisme? E-mail général? Bulletin d’information ?

c. À quelle fréquence est-il partagé? Semaine, mensuelle, etc.

d. Existe-t-il une demande du personnel soignant pour des informations / données sur la prescription d'antibiotiques?

12. Existe-t-il des comités ou des réunions ou des unités spécifiques concernant la prescription desantibiotiques?

a. Qui y est représenté?

b. À quelle fréquence se rencontrent-ils?

c. Existe-t-il une présentation croisée avec d'autres unités / équipes? C'est-à-dire comment l'information est-elle partagée entre les équipes / comités?

Local:

13. Existe-t-il une mesure/évaluation locale régulière de la prescription d'antibiotiques (à l’échelle des services ou des unités)?

a. Si oui, qu'est-ce qui est mesuré?

b. Qui mesure les données?

c. À qui s'adresse le retour de données?

d. À quelle fréquence?

14. Un engagement public / patient dans la prescription de médicaments antimicrobiens est-il entrepris?

15. En ce qui concerne les antibiotiques, quel type de données souhaitez-vous disposer au niveau organisationnel et au niveau du terrain ?

a. Pourquoi ces données particulières?

**B. Interventions individuelles:**

*Les participants seront invités à réfléchir à une* ***intervention récente*** *qu'ils ont mise en œuvre dans le cadre de leur programme de prescription d'antibiotiques et on leur demandera ce qui suit:*

Objectifs et modalités d'intervention

1. Êtes-vous au courant des interventions récentes visant le bon usage d'antibiotique dans votre hôpital?

a. Qu'en est-il des autres hôpitaux?

2. Si OUI, posez les questions ci-dessous,

si NON: existe-t-il des interventions liées aux antibiotiques, qui, selon vous, devraient être mises en œuvre dans votre structure? Si oui, adaptez les questions suivantes en utilisant le futur.

3. Quel a été le déclencheur de l'intervention? Une épidémie de bactérie résistante? Réduction des coûts? AMR?

4. Pourquoi cette intervention serait-elle une amélioration?

5. Quels étaient les objectifs de l'intervention?

a. Qui a participé à la définition des objectifs?

b. Des résultats imprévus potentiels ont-ils été abordés?

6. Comment et quoi avez-vous mesuré?

a. Qui a recueilli ces données?

b. Pourrait-on avoir accès aux données existantes de la pharmacie hospitalière?

Boucle de rétro-information

7. Existe-t-il un système de rétro-information

a. Qui a reçu cette rétro-information

b. À quelle fréquence?

c. Dans quel format?

d. Est-ce qu'il y avait un dialogue structuré? Par exemple, une discussion ou une explication autour des résultats?

8. Y-a-t-il eu un suivi de la rétro-information?

a. Si oui, décrivez-le

Enfin,

Que pensez-vous du fait que la Norvège / France / Inde / BF comparent leurs systèmes de santé concernant la résistance bactérienne et les infections associées aux soins ?

Dans un monde idéal, qu'est-ce que vous voudriez voir implémenter sur la prescription d'antibiotiques?

Quels sont les obstacles à la mise en œuvre?
